# An Analysis of Patients That Underwent Computed Tomography Pulmonary Angiography with the Prediagnosis of Pulmonary Embolism in the Emergency Department

**DOI:** 10.1155/2014/470358

**Published:** 2014-05-15

**Authors:** Engin Ozakin, Filiz Baloglu Kaya, Nurdan Acar, Arif Alper Cevik

**Affiliations:** ^1^Department of Emergency Medicine, Eskisehir Osmangazi University Medical Center, Meselik 26480, Eskisehir, Turkey; ^2^Medical College and Health Sciences, United Arab Emirates University, Al Ain, United Arab Emirates

## Abstract

*Introduction.* The purpose of this study is to analyze the frequency of other diagnoses and findings in patients that were diagnosed with or not diagnosed with PE following the CTPA in the ED and to analyze the relationship between diagnosis and D-dimer.* Instrument and Method.* This study involves all patients that presented to the ED that underwent CTPA with the prediagnosis of PE. The items considered in this study were their reason for presenting to the ED and pretest clinical risks for PE, D-dimer, and CTPA results.* Findings.* Of the 696 cases, the most common cause was shortness of breath (59.3%). The CTPA showed that 145 (20.83%) patients were suffering from PE. Among the remaining cases, 464 (66.66%) patients had pathological findings other than PE and 87 (12.5%) patients were reported as normal. The most common pathological results other than PE found in CTPA were atelectasis in 244 (39.9%) and ground glass in 165 (23.7%), as well as nonpulmonary results in 70 (10.05%) patients. The differences in D-dimer results of patients diagnosed with PE, patients diagnosed with another pathology, and patients with normal CTPA results were statistically significant (*P* < 0.001).* Conclusion.* CTPA scanning, performed on the basis of assessment scoring, helps in discovering other fatal pathologies in addition to PE.

## 1. Introduction

PE is a frequently encountered disease that is difficult to diagnose, and the disease may develop a fatal course [1]. The disease mostly occurs when deep vein thrombosis blocks the pulmonary artery completely or partially. The diversity of symptoms and findings may mask the clinical evidence and cause the disease to be unnoticed. In recent years, there have been improvements in the diagnosis and treatment of the disease. Early diagnosis and treatment are lifesaving. That is why, in patients with suspected PE, risk factors as well as clinical, laboratory and imaging results should be examined carefully. In cases with suspicion, the frequency of PE ranges between 8% and 39% [2, 3]. While the mortality rate of PE is approximately 30% in noncured patients, the rate reduces to 2–8% with treatment [4, 5]. The symptoms and findings such as shortness of breath, chest pain, syncope, hyperventilation, and unexplained tachycardia are not specific to PE and may develop as well in case of pneumonia, acute exacerbation of COPD, malignity, pleural effusion, or cardiac diseases [6]. That is why the most significant phase of PE diagnosis is clinically suspected. Because the specificity of clinical and physical examination in the diagnosis is low, diagnostic tests should support the examination. Although some algorithms and clinical risk rules have been defined for the diagnosis of PE, there is no standard approach concerning the tests or imaging methods that should be requested. The tests used for the diagnosis are arterial blood gas, D-dimer test, electrocardiogram, chest radiography, echocardiography, CTA, pulmonary angiography, magnetic resonance imaging, and ventilation perfusion scintigraphy. Although pulmonary angiography is the gold standard in the diagnosis of PE, it has not been preferred very frequently because it is an invasive method. Computerized tomography pulmonary angiography (CTPA) has been preferred primarily in clinics because it potentially discovers alternative diagnoses [7]. Because the source of emboli is deep veins in the lower extremities, the ability of CTPA to detect PE increases to 90–93% when employed together with CT venography of lower extremities [7, 8]. The aim of this study was to determine the diagnosis of non-PE pathologies in patients admitted to the emergency department and CTPA was studied.

## 2. Method

This study was conducted in the adult emergency department of a university hospital between July 1, 2010, and July 31, 2013, after the permission required was received from the local board of ethics. The study involves patients aged over 18 who underwent CTPA with the prediagnosis of PE. The relevant imaging results were reported by radiology specialists. The demographic information about patients, their reasons for presenting to the ED, clinical symptoms and findings, D-dimer results, and CTPA reports were examined retrospectively. The patients that underwent CTPA due to trauma, aortic dissection, and tumor were not included in the study. The Wells criteria (Table 1) were used for pretest risk classification.

64-detector CT (Aquilion 64, Toshiba Aquillon, Otawara, Japan) was used for all patients. The records in the radiology department assured that, in the process of scanning, all patients were in supine position, the scanning covered the area from the lower part of the lung to lower cervical vertebras, image and volume scanning was 5 mm, and all cases were administered nonionic opaque agent (for patients up to 100 kg 100 mL in the form of 3 mL/s and for patients over 100 kg 100–150 mL/kg) by autoinjectors branded Missouri through the vascular access established on the antecubital vein. Furthermore, the scanning started automatically with a delay period of 12 to 20 seconds, depending on the patient, when the density of pulmonary arteries reached 120 Hounsfield units, and, in order to avoid artifact, the patients received firstly bolus serum physiologic solution, then the opaque agent, and then again bolus serum physiologic solution. The researchers firstly examined, through the reports, the filling defects in the pulmonary arterial system in mediastinal screen images. In the cases without PE, the findings of pulmonary parenchyma, mediastinum, and any pathology related to the cardiovascular system, pleural structures, and the upper abdomen were recorded. In the cases without PE, CT findings and the relationship between these findings and D-dimer results were analyzed. Furthermore, the age, gender, complaints, and rate of admission to the hospital were compared for patients diagnosed with PE, patients not diagnosed with PE, and patients that had normal CTPA results. CT findings and Wells score were analyzed by Pearson's chi-square test. Due to the abnormal distribution, Kruskal-Wallis nonparametric test was used to analyze the relation between Ct findings and D-dimer. *P* < 0.05 was accepted as significance. In patients with CT findings, pathologies were tested by two-proportion *Z* test in PE and non-PE groups. All data were recorded and analyzed in SPSS 20.

## 3. Findings

The study comprises 696 patients, 364 (52.3%) of whom were female and 332 (47.7%) of whom were male. The CTPA results showed that 145 (20.8%) patients had symptoms compatible with PE and 464 (66.7%) patients had symptoms compatible with diseases other than PE. The imaging of 87 (12.5%) patients did not provide any pathological findings. The distribution of demographic and clinical characteristics of cases according to CTPA results is provided in Table 2.

The most common causes for presenting to the ED were as follows: shortness of breath in 350 (50.2%), chest pain in 93 (13.3%), back pain in 32 (4.5%), change in consciousness in 77 (11%), hemoptysis in 25 (3.5%), palpitation in 22 (3.1%), syncope in 26 (3.7%), shortness of breath and chest pain in 14 (2%), back and chest pain in 9 (1.2%), stomach ache in 13 (1.8%), and asthenia in 12 (1.7%) cases. The other causes were nausea, vomiting, vertigo, swelling in extremities, cough, and fever.

According to the risk scoring based on Wells criteria, 239 (34.3%) cases were in the low, 352 (50.6%) cases in the moderate, and 105 (15.1%) cases in the high risk range. The distribution of risk scoring by diagnosis is provided in Table 3, and there is statistically significant difference between groups (*P* < 0.001).

Of 696 patients, 563 underwent D-dimer testing. All patients that did not have a D-dimer testing had moderate or high scores according to the Wells criteria. Wells score is compared with D-dimer results; the following results were obtained: 2971 ± 3571 ng/mL (range: 160–36000) in cases with low risk, 4362 ± 6054 ng/mL (range: 242–36000) in cases with moderate risk, and 9836 ± 10689 ng/mL (range: 805–36680) in cases with high risk.

**Figure 1:**
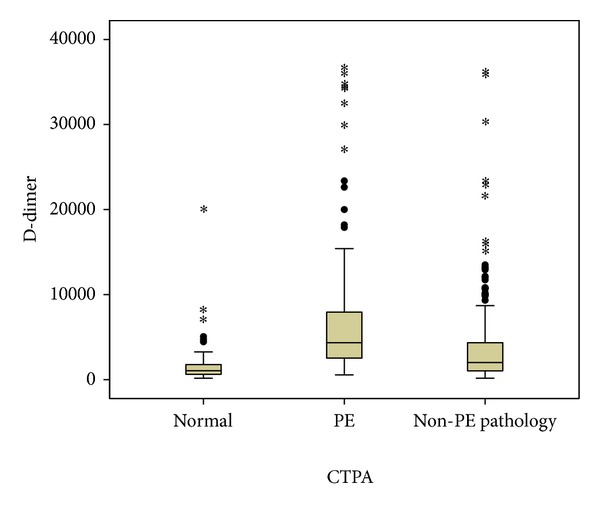
Independent samples Kruskal-Wallis test.

The CTPA results provided three groups of cases: those diagnosed with PE, those diagnosed with a disease other than PE, and those with normal results. The paired comparisons based on the Kruskal-Wallis test showed that the three groups were different from each other with respect to D-dimer results, and the differences are statistically significant (*P* < 0.001) (Table 4, Figure 1).

In the cases diagnosed with PE, the emboli were massive in 22 cases, segmental in 102 cases, and subsegmental in 21 cases. The most common disorders in the cases not diagnosed with PE were atelectasis, ground glass appearance, and pleural effusion. The distribution of CT findings of cases diagnosed and not diagnosed with PE is provided in Table 5.

Following the first examination, tests, and treatment in the emergency department, 128 (93.1%) out of 145 patients diagnosed with PE were transferred to the department of chest diseases, and 219 (47.2%) out of 464 cases diagnosed with another disorder and 14 cases that had normal results in CT were transferred to relevant departments in consideration of the diagnosis. The rates of hospitalization according to CT findings can be seen in Table 6.

## 4. Discussion

PE is a fatal cardiovascular disease which has been encountered frequently. Clinical evidence is important for the diagnosis of PE. 90% of the patients suffer from one or all of the following complaints: shortness of breath, chest pain, back pain, change in consciousness, and syncope [6]. PE should be considered not only in an acute setting but also in patients with prolonged respiratory symptoms [9]. However, these symptoms are not specific to PE and may be seen in the case of diseases other than PE. In the present study, of 464 patients not diagnosed with PE, 238 had shortness of breath, 74 had chest pain, 52 had change in consciousness, and 25 had back pain.

Algorithms incorporating clinical prediction rules and/or D-dimer testing have been developed to guide the evaluation of patients presenting with suspected PE. Two such algorithms, the Wells score coupled with D-dimer testing (Wells/D-dimer), have demonstrated high negative predictive value (NPV) in large prospective emergency department (ED) studies [10].

The risk stratification by using Wells score in non-PE and normal groups could be considered as moderate or high risk. Due to this result, further radiological assessment is essential especially in non-PE patients with similar clinical situations and risk factors.

Although pulmonary angiography is acknowledged as the gold standard for the diagnosis of PE, it has not been used routinely because it is an invasive method and not available in every health center [11]. V/P scintigraphy is useful to determine PE risk before performing the CTPA and has been used for long years as the most preferred method after chest radiography. The disadvantage of V/P scintigraphy is that it does not directly show the clot but detects secondary effects. This method cannot be used to detect pathologies in the pulmonary parenchyma and other mediastinal structures. Today, the technological developments in devices have reinforced the value of CTPA in the diagnosis of PE. The devices are available in many health centers. In the images provided by these devices, fine cross sections of vascular structures may be seen in a detailed way. When required, anatomic structures may be examined three-dimensionally. Particularly in thorax CT scanning, with the use of CT venography, the sensitivity of CTPA to PE increased from 83% to 93% [7, 8]. However, the selection of patients requires specific care because of four significant disadvantages. These are the radiation exposed, the nephrotoxic effect risk of iodized opaque agents, longer period of stay in already crowded emergency departments and thus slower functioning of the emergency departments, and high costs. In case of suspected PE, there is a need to discuss the benefits of risk criteria before imaging. Despite risk scoring scales such as Wells, Geneva, and many studies show that clinical diagnosis rules do not affect the rate of CTPA use for the diagnosis or exclusion of PE [12, 13]. In our study, the Wells scoring was used for clinical risk assessment. The simplified Wells score is accurate in predicting the clinical probability of PE in patients for whom pulmonary embolism was suspected and in predicting that in this population the PE prevalence is higher than other cohorts [14]. The D-dimer test is considered to be highly sensitive, but nonspecific [15]. As a result, the role of this test has typically been limited to rule out a PE in case of low suspicion [15]. Normal range for D-dimer is 0–500 ng/mL. According to the literature, D-dimer values greater than 500 ng/mL are considered positive [15]. Although a normal D-dimer value is used to rule out PE, values greater 500 ng/mL always cannot indicate PE. According to Wells score, 30 patients had D-dimer values lower than 500 ng/mL (low in 18 (60%) patients and moderate in 12(40%) patients). There are many factors that can contribute to the elevation of a D-dimer value. D-dimer can be elevated in pregnancy, trauma, postoperative periods, inflammatory states, renal disease, stroke, myocardial infarction, disseminated intravascular coagulation, and cancer [15]. This situation was the same as in patients without PE with elevation of D-dimer levels. Also it has been found that the D-dimer values are higher in Africo-Americans [16].

In our study, the rate of PE diagnosis is comparable with the rates provided in the literature (8–39%) [2, 7]. The reasons for this may be listed as the use of Wells criteria for risk scoring to detect CTPA indication, the use of D-dimer and arterial blood gas testing, and careful assessment of vital findings and clinical parameters.

In imaging, the radiological findings of patients not diagnosed with PE showed infiltrative lesions such as consolidation and ground glass appearance which may indicate pneumonia and atelectasis, bronchiectasis, and chronic lesions of obstructive pulmonary diseases that show emphysematous changes and pleural effusion.

In some patients already diagnosed with malignity, new lesions were found and new mass formation was seen in the lung. In addition to pulmonary pathologies, fatal situations that change the patient management such as aortic dissection, aortic aneurism, and pericardial effusion were found.

## 5. Limitations

This study was done retrospectively; therefore, required information could not be found in the folder or computer record of some study patients.

The study was conducted at one ED, so the results may not be generalizable to other EDs in other settings. While we examined the use of two common clinical decision rules, Wells score and PERC, there are other commonly used scores such as the Geneva score and Pisa model, which were not evaluated.

## 6. Conclusion

Emergency medicine physicians frequently use clinical diagnosis rules to diagnose PE. They commonly perform CTPA. CTPA, a quick and accurate procedure that is accessible on a 24 h basis in most EDs, is a valuable tool for the diagnosis of PE. CTPA not only ensures that PE is noticed but also provides distinctive results for the diagnosis of other pathologies. Our study also shows that CTPA scanning, performed on the basis of assessment scoring, helps in discovering other fatal pathologies in addition to PE. That is why prediction rules such as Wells and Geneva may be revised to cover criteria for the diagnosis of PE and alternative thorax pathologies through CTA.

## Figures and Tables

**Table:** (a)

Criterion	Points
Suspected DVT	3.0
An alternate diagnosis is less likely than PE	3.0
Heart rate > 100 beats/min	1.5
Immobilization or surgery in the previous four weeks	1.5
Previous DVT/PE	1.5
Hemoptysis	1
Malignancy (on treatment, treated in past six months)	1

**Table:** (b)

Score range	Mean probability of PE	%with score	Interpretation of risk
0–2 points	3.6%	40%	Low
3–6 points	20.5%	53%	Moderate
>6 points	66.7%	7%	High

Source: [17].

**Table 2:** Demographic and clinical characteristics according to CT results.

	PE	Non-PE pathology	Normal	Total
Age	63.05 (±17.52, 29–92)	66.45 (±15.96, 21–93)	52.44 (±19.55, 20–88)	63.17 (±16.82, 20–93)
Male	61 (18.4%)	241 (72.6%)	30 (9%)	332 (100%)
Female	84 (23.1%)	223 (61.3%)	57 (15.7%)	364 (100%)

**Table 3:** CT findings—Wells score.

CT findings	Low	Moderate	High
Pulmonary embolism	**15 **	**103**	**27**
Massif	0	7	15
Segmental	6	90	6
Subsegmental	9	6	6
Non-PE pathology	**176**	**217**	**71**
Normal	**48**	**32**	**7**

Total	**239**	**352**	**105**

Pearson chi-square test = 123,439, *sd*⁡ = 6, and *P* < 0.001.

**Table 4:** CT findings—D-dimer.

CT findings	D-dimer test (*N*)	Median D-dimer 25–75% (range)	
Normal	77	1038 (614, 1826)	*P* < 0.001
Non-PE pathology	340	2010 (1030, 4342)	
PE	145	4342 (2510, 8471)	

Total	562		

Kruskal-Wallis test is used.

**Table 5:** CT findings and frequencies in PE and non-PE patients.

BT findings	PE (%)	Non-PE (%)	Total	
Consolidation	6 (4.1)	83 (17.9)	89	*P* < 0.001
Ground glass opacification	18 (12.4)	165 (35.3)	183	*P* < 0.001
Bronchiectasis	6 (4.1)	48 (10.3)	54	*P* = 0.004
Atelectasis	34 (23.4)	244 (52.5)	278	*P* < 0.001
Fibrosis	2 (1.3)	52 (11.2)	54	*P* < 0.001
Emphysema	5 (3.4)	96 (20.6)	101	*P* < 0.001
Lymphadenopathy	3 (2)	39 (8.4)	42	*P* < 0.001
Pleural effusion	22 (15.1)	161 (34.6)	183	*P* < 0.001
Mass	2 (1.3)	29 (6.2)	30	*P* < 0.001
New	—	14 (2)		
Old	2 (1.3)	14 (2)		
Metastatic lesion	1 (0.6)	11 (2.3)	11	*P* = 0.088
New	1 (0.1)	3 (0.4)		
Old	—	8 (1.1)		
Other				
Pulmonary HT	4 (2.7)	19 (4.0)	23	*P* = 0.416
Cardiomegaly	—	8 (1.1)	8	
Pericardial effusion	6 (4.1)	10 (2.1)	16	*P* = 0.267
Aortic aneurysm	—	22 (3.2)	22	
Aortic dissection	—	9 (1.3)	9	
Pneumothorax	—	7 (1)	7	
Herniation	—	1 (0.1)	1	
Bullae	—	1 (0.1)	1	
Hepatic cyst	—	1 (0.1)	1	

Two-proportion *Z* test is used.

**Table 6:** Final decision—CT findings.

	PE (%)	Non-PE pathology (%)	Normal (%)
Service	78 (53.8)	103 (22.2)	5 (5.7)
Intensive care unit	60 (41.4)	116 (25)	9 (10.3)
Refuse treatment	2 (1.4)	1 (0.2)	0
Discharge	5 (3.4)	244 (52.6)	73 (83.9)

Total	145	464	87
